# Precision management of cenobamate in drug-resistant epilepsy: integrating pharmacogenetics, therapeutic drug monitoring, and real-world clinical strategies

**DOI:** 10.3389/fphar.2026.1830217

**Published:** 2026-06-04

**Authors:** Giuseppe d’Orsi, Maria Teresa Di Claudio, Alessia Cafaro, Sebastiano Barco, Marta Robustella, Nicolò Locatelli, Antonella Liantonio, Paola Imbrici, Grazia Ciavarella, Antonio Rinaldi, Giuseppe Fania, Umberto Costantino, Massimo Carella, Giuliana Cangemi, Giuseppe Miscio

**Affiliations:** 1 Neurology Unit - Epilepsy Center, Fondazione IRCCS Casa Sollievo Della Sofferenza, San Giovanni Rotond, Italy; 2 Biochemistry, Pharmacology and Newborn Screening Unit, Central Laboratory of Analyses, IRCCS Istituto Giannina Gaslini, Genova, Italy; 3 Department of Pharmacy - Drug Sciences, University of Bari “Aldo Moro”, Bari, Italy; 4 Medicina Trasfusionale e Laboratorio Analisi, Fondazione IRCCS Casa Sollievo Della Sofferenza, San Giovanni Rotondo, Italy; 5 UOC Genetica Medica, Fondazione IRCCS Casa Sollievo Della Sofferenza, San Giovanni Rotondo, Italy

**Keywords:** cenobamate, pharmacogenetics, drug-resistant epilepsy, real-world evidence, therapeutic drug monitoring, precision medicine

## Abstract

Cenobamate (CNB) demonstrates high efficacy in drug-resistant focal epilepsy, yet optimal management requires personalized strategies. This exploratory case series describes five patients selected from a cohort of 125 individuals treated with CNB, examining the relationship between pharmacogenetic (PGx) profiles and clinical outcomes. Clinical data and therapeutic drug monitoring (TDM) were integrated with targeted Next-Generation Sequencing of key metabolic genes (UGT2B7, UGT2B4, CYP2E1, CYP2B6, CYP2A6, CYP3A4, CYP2C19) to predict metabolizer phenotypes. Three patients (Cases 1, 3, 4) achieved seizure freedom or ≥50% response at sub-target or target doses (100–200 mg/day*)* enabling early de-escalation of concomitant sodium channel blockers and valproate. This success was supported by a proactive digital communication protocol and a mandatory 100 mg clinical checkpoint. Case 2—a non-responder at 400 mg/day despite therapeutic plasma levels (28.22 mg/L)—exhibited a predicted “Ultrarapid Metabolizer” (UM) phenotype characterized by UGT2B7 Haplotype 4 and CYP2E1 duplication, suggesting possible pharmacokinetic contribution to non-response. Case 5 experienced dose-limiting toxicity at 100 mg/day; we hypothesized this toxicity could be driven by a drug-drug-gene interaction involving CNB-mediated *CYP2C19* inhibition that impairs clearance of N-desmethylclobazam (the active metabolite of clobazam) in a patient with impaired *CYP2A6* activity and intermediate *UGT2B7* function. As an exploratory case series, these findings require validation in adequately powered prospective studies. Associations between UM phenotype and non-response, and between PM phenotype and dose-limiting toxicity, represent preliminary observations that may reflect pharmacokinetic variability. Nevertheless, our experience is consistent with a personalized, sub-target dose titration strategy that may help minimize adverse events in complex polypharmacy settings.

## Introduction

Drug-resistant epilepsy affects approximately 30% of patients despite multiple antiseizure medications (ASMs). Cenobamate (CNB), with dual GABAergic and sodium channel mechanisms, has demonstrated exceptional efficacy in randomized controlled trials, with seizure freedom rates reaching 20%–25% in highly refractory populations (Nakamura et al., 2019; Krauss et al., 2020). However, real-world implementation reveals substantial interindividual variability in therapeutic response and tolerability (Chung et al., 2020; Sperling et al., 2020). CNB exhibits linear pharmacokinetics and is primarily metabolized via UGT2B7-mediated glucuronidation (∼70–80%), with secondary contributions from UGT2B4 and oxidative pathways (CYP3A4, CYP2E1, CYP2B6, CYP2C19, CYP2A6) (Nakamura et al., 2019; Patsalos and Perucca, 2003; Vernillet et al., 2020). Despite this well-characterized metabolic profile, clinical practice reveals puzzling scenarios: patients achieving seizure freedom at 100 mg/day, while others show no response at 400 mg/day despite therapeutic plasma levels, and still others experiencing dose-limiting toxicity at minimal doses. Pharmacogenetics (PGx) has transformed personalized medicine in oncology and psychiatry (Kirchheiner et al., 2005; Relling and Evans, 2015), yet its application to newer ASMs like CNB remains limited (Tate and Goldstein, 2004; Sisodiya and Cavalleri, 2012). While therapeutic drug monitoring (TDM) is increasingly recognized as valuable for CNB (Kwan et al., 2010; Pigliasco et al., 2024), no studies have systematically integrated TDM with comprehensive pharmacogenetic profiling. Drawing from our epilepsy center’s experience with 125 patients treated with CNB, we present five representative cases illustrating the spectrum of clinical outcomes and their potential genetic underpinnings. We implemented two key safety strategies: (Nakamura et al., 2019): a mandatory clinical checkpoint at 100 mg, and (Sharma et al., 2021) proactive digital communication protocols. We integrated clinical outcomes with TDM and targeted Next-Generation Sequencing of seven key metabolic genes (UGT2B7, UGT2B4, CYP2E1, CYP2B6, CYP2A6, CYP3A4, CYP2C19). We hypothesized that: (Nakamura et al., 2019): patients with ultrarapid-metabolizer (UM) phenotypes would exhibit therapeutic failure due to accelerated drug clearance; (Sharma et al., 2021); patients with poor-metabolizer (PM) phenotypes would experience dose-limiting toxicity; and (Perucca et al., 2023) integration of PGx and TDM could enable precision-guided CNB therapy.

## Methods

### Study population and case selection

This retrospective, exploratory case series was drawn from the Epilepsy Center at Fondazione IRCCS Casa Sollievo della Sofferenza, San Giovanni Rotondo, Italy. During the study period (January 2022 – December 2025), 125 patients initiated CNB therapy at our center. Of these, 91 patients with focal drug-resistant epilepsy meeting ILAE criteria (Kwan et al., 2010) and with adequate follow-up (minimum 3 months) comprised the source cohort for this analysis. The remaining 34 patients were excluded due to generalized or combined epilepsy syndromes (9 patients), insufficient follow-up duration (12 patients), or incomplete baseline data (13 patients). The source cohort (n = 91) had a median age of 37 years (IQR: 27–50.3), with 55% female. The median age at epilepsy diagnosis was 11 years (IQR: 5–22). Overall, patients exhibited high treatment resistance, having failed a mean of 7.42 ASMs (range: 1–21), and were on a mean of 2.27 concomitant ASMs (range: 1–5) at baseline. Pre-CNB seizure burden was substantial, with a mean of 28.77 seizures in the month before CNB initiation (85.87 seizures in the 3 months before CNB). From this cohort, five clinical vignettes were purposively selected based on: (Nakamura et al., 2019): spectrum of clinical outcomes (seizure freedom, ≥50% response, non-response, early discontinuation due to adverse events); (Sharma et al., 2021); availability of complete data including therapeutic drug monitoring (TDM) and informed consent for comprehensive genetic testing; and (Perucca et al., 2023) illustrative value for management strategy evolution. These cases represent the clinical diversity observed in our practice: two patients achieving seizure freedom (Cases 1 and 4), one responder with ≥50% seizure reduction (Case 3), one non-responder despite maximum dosing (Case 2), and one early discontinuation due to dose-limiting toxicity (Case 5).

### Clinical data collection

Clinical data were extracted from electronic medical records by two independent investigators (GdO, MTD). Parameters included baseline characteristics, titration schedule, safety monitoring via Proactive Communication Protocol, and efficacy outcomes categorized as seizure freedom (100% reduction ≥3 months), responder (≥50% reduction), or non-responder (<50% reduction).

For the purposes of this study, we define doses as follows: “sub-target dose” refers to doses below the recommended maintenance dose of 200 mg/day (i.e., ≤150 mg/day, corresponding to the titration phase per FDA labeling); “target dose” refers to 200 mg/day; and “high dose” refers to doses above 200 mg/day (up to the maximum of 400 mg/day). The term “low dose” is used specifically to indicate 100 mg/day, which corresponds to weeks 7–8 of the standard titration schedule and represents the dose at which our mandatory clinical checkpoint is performed.

### Therapeutic drug monitoring

CNB plasma concentrations were measured at steady-state using HPLC-MS/MS (Pigliasco et al., 2024). Concomitant ASM concentrations (LEV, VPA, TPM, LTG, LCM, ZNS, CBZ and metabolites) were determined using MassTox® TDM Series A (Chromsystems).

### Pharmacogenetic analysis

Targeted NGS was performed using SureSelect XT HS2 (Agilent) with custom-designed probes covering UGT2B7, UGT2B4, CYP2E1, CYP2A6, CYP2B6, CYP3A4, and CYP2C19. Library preparation used 100 ng input DNA, enzymatic fragmentation, and Illumina MiSeq sequencing (2 × 150 bp). Variants were confirmed by Sanger sequencing and/or Real-Time PCR.

Genotype-to-phenotype translation was performed at the gene level using published functional evidence and gene-specific interpretation rules. For genes with established clinical frameworks, standardized nomenclature and phenotype translation were applied; for genes without fully standardized frameworks, phenotype assignments were based on the totality of available literature and functional characterization data (Supplementary Tables S1, S2) (Desta et al., 2021). In particular, *CYP2B6* phenotypes were translated according to CPIC guidelines, whereas *UGT2B7*, *UGT2B4, CYP2E1*, and *CYP2A6* phenotypes were inferred from published functional studies (Yong et al., 2011, Fairbrother et al., 1998, Tanner and Tyndale, 2017, Innocenti et al., 2008; Kwara et al., 2009, Tremmel et al., 2016, Huang et al., 2012, Langlois et al., 2024, Oscarson et al., 2002, Fukami et al., 2005; Han et al., 2012, Hu et al., 2014). When multiple variants or haplotypes were present, the predicted phenotype for each gene was assigned according to the functional effect of the detected allele combination. Because no standardized framework exists for cross-gene composite phenotype calls in this setting, the final NM/UM/PM label was assigned using our interpretation framework, integrating the direction of effect across the panel and, at the same time drawing our conclusions. The molecular data and the functional effects of the identified gene variants were contextualized with plasma level measurements and the patients’ seizure outcomes. This approach was exploratory and intended to support hypothesis generation rather than to establish a cross-gene translation standard.

### Ethical approval

The study was approved by Comitato Etico Territoriale Regione Puglia (Prot. N. 712, Nov 05, 2025; Study number 7910). Consent for publication was obtained.

### Terminology and definitions

Dose categories: “sub-target dose” refers to doses ≤150 mg/day (corresponding to the titration phase per FDA labeling); “target dose” refers to 200 mg/day; “high dose” refers to doses above 200 mg/day (up to the maximum of 400 mg/day).

Clinical assessment: the “100 mg clinical checkpoint” refers to the mandatory clinical evaluation performed when patients reach 100 mg/day.

Metabolic phenotypes: Poor Metabolizer (PM), Intermediate Metabolizer (IM), Normal Metabolizer (NM), and Ultrarapid Metabolizer (UM), classified according to CPIC nomenclature where available and published functional studies for genes without CPIC guidelines.

Efficacy outcomes: seizure freedom (100% seizure reduction sustained ≥3 months), responder (≥50% seizure reduction), and non-responder (<50% seizure reduction).

## Results

**FIGURE 1:**
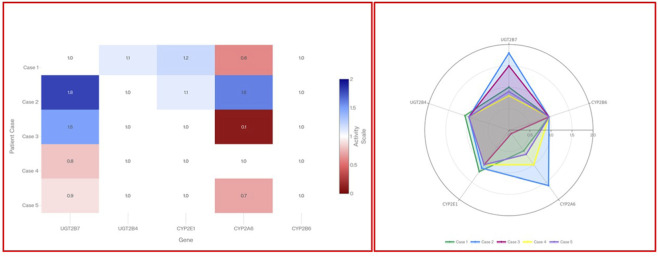
Integrated Genomic and Metabolic Profiling. Left: Heatmap of relative enzymatic activities in cenobamate metabolism. A thermal representation of normalized enzymatic activities (scale 0–2) across the five patients. The color gradient ranges from blue (hyperactivity, >1) to white (normal activity, ≈1) and red (hypoactivity, <1). Key patterns include: Prevalent hyperactivity in Case 2 (UM phenotype, indicated by blue tones for *UGT2B7* and *CYP2A6*). Marked hypoactivity for *CYP2A6* in Case 3 (deep red). Standard profiles in Cases 4 and 5 (predominantly white). Color Scale: 0 (dark red, severe loss-of-function) to 2 (dark blue, significant overexpression). Right: Radar chart of relative enzymatic activities in cenobamate metabolism. The radar chart summarizes the normalized functional activities (scale 0–2) of *UGT2B7, UGT2B4, CYP2E1, CYP2A6*, and *CYP2B6* in five patients with ultra-refractory focal epilepsy. Each axis represents a specific gene (1 = reference normal activity; >1 = hyperactivity; <1 = hypoactivity). The distinct polygons highlight the inter-patient variability across the metabolic pathways. These two figures provide a dual perspective on the cohort’s genomic landscape: the radar chart emphasizes inter-patient differences per gene, while the heatmap highlights inter-gene differences per patient.

Five patients with refractory focal epilepsy were purposively selected as representative clinical vignettes; their detailed features, outcomes, and PGx findings are summarized in Table 1. The key takeaways and refined management strategies for CNB are outlined in Tables 2, 3; Figure 1 provide a comprehensive summary of PGx findings, correlating specific genetic haplotypes with metabolic phenotypes. The molecular findings and the functional impact of the detected variants were interpreted in the context of plasma drug concentrations and the observed clinical response in seizure control. Specifically, gene-level phenotype assignments were not considered in isolation, but were integrated with therapeutic drug monitoring and the patients’ efficacy or tolerability outcomes to contextualize the final NM, UM, or PM designation.

**TABLE 1:** Summary of patient features and outcomes.

​	Case 1	Case 2	Case 3	Case 4	Case 5
Age / Sex	24/Female	39/Male	25/Male	21/Female	26/Male
Etiology	Structural	Structural	Neoplastic	Unknown	Unknown
Prior Failed ASMs (n)	8 (CBZ, LTG, LEV, LCM, OXC, BRV, VPA, CLB)	10 (CBZ, PB, LTG, LEV, TPM, LCM, OXC, BRV, VPA, CLB)	11 (VPA, PB, LTG, ZNS, PER, TPM, LEV, CLB, OXC, RUF, CBZ)	3 (VPA, PER, LTG)	10 (VPA, CBZ, PB, LTG, PER, TPM, LEV, CLB, OXC, BRV)
Seizure Frequency	Pluridaily / Daily	Pluridaily / Daily	Pluri-monthly	Pluri-monthly	Pluri-monthly
Baseline Treatment	LEV 1000 mg/day + VPA 500 mg/day	TPM 500 mg/day + RUF 200 mg/day + Delorazepam 2 mg/day	VPA 1000 mg/day + LTG 150 mg/d	LEV 2000 mg/day + LCM 400 mg/day	CBZ RM 1200 mg/day + ZNS 400 mg/day + CLB 30 mg/day
CNB Strategy Focus	Anticipated Titration Checkpoint	Maximal Titration	Polypharmacy Reduction	Early Intervention	Mitigation of Drug-Drug-Gene Interaction
Final CNB Dose	100 mg/day	400 mg/day	150 mg/day	100 mg/day	100 mg/day
Primary Outcome	Sustained Seizure Freedom	Non-Responder	>50% Reduction	Sustained Seizure Freedom	Discontinuation due to AEs
Tolerability	Good	Good	Good	Good	Poor (Somnolence, Vertigo)
PGx Phenotype	Normal Metabolizer	Ultra-Metabolizer	Normal Metabolizer	Normal Metabolizer	Poor Metabolizer
Key Lesson	Efficacy at minimal dose validates the 100 mg checkpoint strategy.	Non-response likely due to rapid drug clearance (PK failure), not pharmacodynamic resistance.	Effective response allowed VPA discontinuation and LTG reduction, improving holistic care.	Safety protocols built confidence for earlier use in the drug-resistance course.	Toxicity due to CNB-mediated phenoconversion of CLB in a patient with PM variants.

This table summarizes the demographic characteristics, clinical history, treatment regimens, and pharmacogenetic (PGx) profiles of five representative patients treated with Cenobamate. It highlights the clinical trajectory from ultra-refractory cases to earlier interventions, illustrating the relationship between metabolic phenotypes (NM, UM, PM) and clinical outcomes such as seizure freedom, pharmacokinetic failure, or dose-limiting toxicity. Abbreviations: AEs: adverse events; ASM: anti-seizure medication; BRV: brivaracetam; CBZ: carbamazepine; CLB: clobazam; CNB: cenobamate; LCM: lacosamide; LEV: levetiracetam; LTG: lamotrigine; NM: normal metabolizer; OXC: oxcarbazepine; PB: phenobarbital; PER: perampanel; PK: pharmacokinetic; PM: poor metabolizer; RUF: rufinamide; TPM: topiramate; UM: Ultrarapid Metabolizer; VPA: valproate; ZNS: zonisamide.

**TABLE 2:** Summary of Evolved Cenobamate Management Strategies and Key Clinical Insights.

​	Key Point / Strategy	Description and Relevance
Initial RWE Strategies	100 mg Anticipated Titration Checkpoint	Utilizing 100 mg as the first therapeutic checkpoint to assess tolerability and initial response (instead of the standard 200 mg). Crucial for enhancing safety, retention, and identifying lower effective doses.
​	Proactive Communication Protocol	Establishing immediate communication channels (WhatsApp, email, phone) for rapid management of potential Adverse Events (AEs). Essential for minimizing dropout and understanding the drug's real-world profile.
Efficacy and Optimal Dosing	Efficacy at Lower Doses	Clinical evidence that substantial response (Seizure Freedom or ≥ 50% reduction) can be achieved at doses ≤ 150 mg/day (Cases 1 and 4), well below the traditional 200 mg threshold.
​	Role in Polypharmacy Reduction	CNB facilitates the de-escalation of concomitant ASMs. Effective response (Case 3) allowed for the discontinuation of VPA Chrono and significant reduction of LTG, improving cognitive outcomes and holistic care.
Pharmacogenetics and Non-Response	Ultra-Metabolizer (UM) Phenotype	Lack of therapeutic response and AEs at maximal doses (400 mg) in an ultra-refractory patient (Case 2) was linked to an UM phenotype involving variants in UGT2B7, CYP2A6, and CYP2E1.
​	Pharmacokinetic Failure	PGx data suggest that some treatment failures are due to insufficient systemic exposure (rapid drug clearance) rather than inherent pharmacodynamic resistance. Highlights the role of genetic screening in non-responders.
Pharmacogenetics and Toxicity	Risk of Drug-Drug-Gene Interaction (Case 5)	Dose-limiting AEs occurred at 100 mg in a patient with a reduced-efficiency phenotype (UGT2B7, CYP2A6). Toxicity resulted from CNB-mediated inhibition of CYP2C19, which elevated levels of the sedating metabolite of Clobazam (CLB).
​	Proactive Genetic Consideration	This case underscores the need to proactively consider PGx to mitigate toxicity, especially when CNB alters the functional metabolic phenotype of co-administered ASMs (phenoconversion).
Evolution of Patient Selection	Expanded Therapeutic Phenotype	Increased confidence from safety protocols allowed for the earlier introduction of CNB in patients with less extreme drug resistance (Case 4, failed only 3 prior ASMs).
​	CNB as an Earlier Option	Transition of CNB from a “rescue medication” for ultra-refractory cases to a viable, earlier option aimed at achieving early and sustained seizure freedom.

This table outlines the transition from standard titration protocols to a personalized approach guided by Real-World Evidence (RWE) and Pharmacogenetics (PGx). It highlights dosing refinements, the impact on polypharmacy reduction, and the critical role of metabolic phenotypes—such as Ultra-Metabolizer (UM) and Poor Metabolizer (PM)—in identifying the underlying causes of treatment failure (pharmacokinetic vs. pharmacodynamic) or dose-limiting toxicity. Abbreviations: AE: Adverse Event; ASM: Anti-Seizure Medication; CLB: Clobazam; CNB: Cenobamate; DRE: Drug-Resistant Epilepsy; LTG: Lamotrigine; PGx: Pharmacogenetics; RWE: Real-World Evidence; VPA: Valproate.

**TABLE 3:** Summary of targeted Next-Generation Sequencing (NGS) of key metabolic genes in five representative clinical cases.

Patient	UGT2B7	UGT2B4	CYP2E1	CYP2A6	CYP2B6	Observed Phenotype
Case 1	NM_001074.4:c.-900AA**#**, rs7438135, (+ 5 additional SNPs: (also known as Haplotype II) (Hu et al., 2014).	NM_021139.3:c.1374TG, p.(Asp458Glu), rs13119049; mild activity increase in homozygous carriers (Yong et al., 2011)	NM_000773.4:c.-71TT, rs6413420; mild transcription/ activity increase (Fairbrother et al., 1998)	Diplotype *2/*9, reduced-activity genotype (Tanner and Tyndale, 2017)	Diplotype *1/*5, normal activity	NM (Normal Metabolizer)
Case 2	NM_001074.4:c.722-314AG, rs62298861; c.735AG, p.(Tyr245=), rs28365062; c.1062CT, p.(Tyr354=), rs4348159 (also known as Haplotype 4): Enhanced expression/ activity (Innocenti et al., 2008; Kwara et al., 2009) + Haplotype II (see above, Case 1).	No functional variants (normal)	Gene duplication with dosage-insensitive compensation (Tremmel et al., 2016); NM_000773.4:c.-333AT, rs2070673, heterozygous mild transcriptional increase (Huang et al., 2012)	Diplotype *46/*46 (3'UTR conversion); increased expression/activity (Langlois et al., 2024)	Diplotype *1/*5, normal activity	UM (Ultra-Rapid Metabolizer)
Case 3	NM_001074.4:c.722-314AG, rs62298861; c.735AG, p.(Tyr245=), rs28365062; c.1062CT, p.(Tyr354=), rs4348159 (also known as Haplotype 4): Enhanced expression/ activity (Innocenti et al., 2008; Kwara et al., 2009) + Haplotype II (see above, Case 1).	No functional variants (normal)	No functional variants (normal)	Diplotype *12/*12 (Hybrid allele CYP2A6-CYP2A7 in homozygosity): severely reduced activity (>90%) (Oscars et al., 2002)	Diplotype *1/*2, normal activity	NM (Normal Metabolizer)
Case 4	NM_001074.4:c.-900GG, rs7438135, homozygous; reduction activity according to (Hu et al., 2014)	No functional variants (normal)	No functional variants (normal)	No functional variants (normal)	Diplotype *1/*1, normal activity	NM (Normal Metabolizer) — least polymorphic patient
Case 5	NM_001074.4:c.-900GA, rs7438135, heterozygous; mild reduction activity	No functional variants (normal)	No functional variants (normal)	Diplotype *1/*18 (reduced catalytic efficiency/substrate recognition) (Tanner and Tyndale, 2017; Fukami et al., 2005; Han et al., 2012)	Diplotype *1/*1, normal activity	PM (Poor Metabolizer) + ADR

The table illustrates the genetic variability of the enzymes involved in the metabolism of Cenobamate (CNB). *UGT2B7* and *UGT2B4*: These enzymes are responsible for the primary metabolic pathway of CNB (glucuronidation). The presence of Haplotype 4 and Haplotype II in Case 2 correlates with an ultra-rapid metabolizer (UM) status, leading to pharmacokinetic resistance. *CYP2E1* and *CYP2A6*: Involved in the oxidative metabolism of CNB. In Case 2, the gene duplication of *CYP2E1* and increased expression of *CYP2A6* contribute to high clearance. Conversely, the hybrid allele in Case 3 and the Y392 F variant in Case 5 lead to severely reduced activity.

Nomenclature note: We refer to two distinct UGT2B7 haplotypes: (a) Haplotype 4 (coding region: rs62298861, rs28365062, rs4348159), associated with enhanced enzyme activity; and (b) Haplotype II (promoter region: rs7438135 c.-900A), associated with increased transcriptional efficiency.

### Case 1: sub-target dose responder with normal metabolizer (NM) phenotype

Clinical Observation: A 24-year-old female with structural focal epilepsy due to lissencephaly (DYNC1H1 mutation) experiencing pluridaily seizures since age four, ultra-refractory after failing 8 ASMs. At CNB initiation, concomitant therapy included LEV 1000 mg/day and VPA Chrono 500 mg/day. CNB was maintained at the sub-target dose of 100 mg/day following the 100 mg clinical checkpoint, achieving sustained seizure freedom at the sub-target dose of 100 mg/day (corresponding to the titration checkpoint) over 32 months.

Biological Interpretation: PGx Analysis: UGT2B7 Haplotype II (c.-900A/A, enhanced transcription), heterozygous UGT2B4 p. Asp458GLu, homozygous CYP2E1 c.-71G>T (increased activity), CYP2A6 2/9 (reduced capacity), CYP2B6 1/1, and CYP2C19 2/2 (PM phenotype). Overall phenotype: Normal Metabolizer (NM), as reduced CYP2A6 was offset by preserved UGT2B7/CYP2E1 function. CYP2C19 PM status had no clinical impact, confirming its minor role in CNB metabolism.

TDM: CNB 12.28 mg/L; LEV 6.34 mg/L; VPA 41.39 mg/L

Clinical Interpretation: Ultra-refractory patients can achieve seizure freedom at minimum dose when metabolic pathways are balanced. The 100 mg checkpoint proved critical in identifying this low-dose responder.

### Case 2: non-responder at high dose w0069th ultrarapid metabolizer (UM) phenotype

Clinical Observation: A 39-year-old male with structural focal epilepsy and pluridaily seizures since age seven, highly ultra-refractory after failing 10 ASMs. At CNB initiation, concomitant therapy included TPM 500 mg/day, RUF 200 mg/day, and delorazepam 2 mg/day. CNB was maximally titrated to 400 mg/day due to persistent lack of efficacy. After 14 months, the patient remained a non-responder with no adverse events.

Biological Interpretation: PGx Analysis: UGT2B7 Haplotype 4 (rs62298861, rs28365062, rs4348159) plus Haplotype II (c.-900A/A), suggesting markedly enhanced glucuronidation (196% higher clearance reported in carriers). CYP2E1 gene duplication (three copies) plus promoter variant rs2070673. CYP2A6 46/46 diplotype (58 bp gene conversion linked to increased mRNA expression) plus five additional enhancing variants. CYP2B6 1/5 (normal metabolizer) (Tanner et al., 2018). Overall phenotype: Ultrarapid Metabolizer (UM) with enhanced activity across UGT2B7, CYP2A6, and CYP2E1.

TDM: CNB 28.22 mg/L (highest in series); TPM 7.96 mg/L; RUF 4.04 mg/L

Clinical Interpretation: Despite achieving 28.22 mg/L plasma level, no clinical response occurred. The predicted UM phenotype raises the hypothesis that enhanced hepatic clearance may contribute to inadequate biophase concentrations despite apparently adequate plasma levels; however, pharmacodynamic resistance at the target-site level represents an equally plausible explanation. This observation raises questions about TDM interpretation in patients with extreme predicted metabolic phenotypes, but remains speculative without direct biophase measurements. The observed dissociation between plasma level (28.22 mg/L — the highest in our series, though no validated therapeutic range for CNB has been established) and lack of efficacy could equally reflect pharmacodynamic resistance.

### Case 3: moderate-dose responder demonstrating gene-gene interaction (NM phenotype)

Clinical Observation: A 25-year-old male with focal epilepsy secondary to anaplastic oligodendroglioma, ultra-refractory after failing 11 ASMs (highest in series). At CNB initiation, concomitant therapy included VPA Chrono 1000 mg/day and LTG 150 mg/day. The patient achieved >50% seizure reduction at the sub-target dose of 150 mg/day, allowing complete VPA discontinuation and LTG reduction to 100 mg/day.

Biological Interpretation: PGx Analysis: UGT2B7 identical to Case 2 (Haplotype 4 + Haplotype II), predicting enhanced glucuronidation. However, CYP2A6 12/12 genotype (hybrid CYP2A6-CYP2A7 gene) causes near-complete loss of oxidative function in homozygotes. Other genes showed no significant variants. Overall phenotype: Normal Metabolizer (NM), based on near-complete CYP2A6 loss counterbalancing enhanced UGT2B7 activity.

TDM: CNB 14.84 mg/L; VPA 90.68 mg/L; LTG 5.68 mg/L.

Clinical Interpretation: This case is consistent with a possible gene-gene interaction. Despite sharing UGT2B7 enhancing haplotypes with non-responder Case 2, this patient achieved efficacy at moderate dose. The critical difference—homozygous CYP2A6*12* causing near-complete oxidative loss—offset enhanced glucuronidation, resulting in balanced clearance. This observation suggests that multi-gene panel assessment may be important for accurate phenotype prediction.

### Case 4: sub-target dose responder with wild-type normal metabolizer (NM) phenotype

Clinical Observation: A 21-year-old female with focal epilepsy of unknown cause, notably shorter drug resistance history (failed only 3 ASMs: VPA, LEV, LCM). At CNB initiation, concomitant therapy included LEV 2000 mg/day and LCM 400 mg/day. The patient achieved sustained seizure freedom at the sub-target dose of 100 mg/day over 15 months without adverse events.

Biological Interpretation: PGx Analysis: UGT2B7 Haplotype I (c.-900G/G, reduced promoter activity). All other genes (UGT2B4, CYP2E1, CYP2A6 1/1, CYP2B6 1/1, CYP2C19, CYP3A4) showed no functional variants—the least polymorphic profile in the series. Overall phenotype: Normal Metabolizer (NM), potentially trending toward Normal/Intermediate.

TDM: CNB 3.8 mg/L; LEV 38.53 mg/L; LCM 12.25 mg/L (mildly elevated but tolerated).

Clinical Interpretation: This case represents evolution from ultra-refractory rescue to earlier intervention. Favorable response at minimal dose with wild-type metabolic profile suggests patients without significant genetic polymorphisms may be ideal candidates for early CNB introduction at conservative doses.

### Case 5: dose-limiting toxicity with suspected drug-drug-gene interaction (PM phenotype)

Clinical Observation: A 26-year-old male with drug-resistant focal epilepsy of unknown etiology, having failed 10 ASMs. At CNB initiation, concomitant therapy included CBZ RM 1200 mg/day, ZNS 400 mg/day, and CLB 30 mg/day. Recognizing CNB-clobazam interaction, CLB was proactively reduced to 20 mg/day. However, within 2 weeks of reaching CNB 100 mg/day, marked somnolence and intense vertigo led to CNB discontinuation.

Biological Interpretation: PGx Analysis: UGT2B7 Haplotype I homozygosity (c.-900G/G, reduced promoter activity). CYP2A6 1/*18 genotype; the* 18 allele (p.Tyr392Phe) affects substrate recognition and lowers enzymatic efficiency. Other genes showed no significant variants. Overall phenotype: Poor Metabolizer (PM) phenotype.

TDM: CNB 8.16 mg/L; CBZ 7.62 mg/L; CBZ-diol 3.26 mg/L; CBZ-epoxide 1.61 mg/L; ZNS 20.57 mg/L. Notably, N-desmethylclobazam (nCLB) was not measured.

Clinical Interpretation: Dose-limiting toxicity at 100 mg/day despite relatively low CNB level (8.16 mg/L) may have been associated with CNB’s CYP2C19 inhibition impairing N-desmethylclobazam clearance. Underlying genetic variants (UGT2B7 Haplotype I, CYP2A6*18*) further reduced CNB clearance, consistent with a hypothesized ‘triple hit’ scenario.

Absence of nCLB measurement is a limitation; this mechanism remains hypothetical.

### Summary of pharmacogenetic-clinical correlations

An exploratory pattern was observed suggesting possible associations between metabolic phenotype and clinical outcome. Cases 1, 3, and 4 (all NM) achieved seizure freedom or ≥50% response at low-to-moderate doses (100–150 mg/day) with CNB levels 3.8–14.84 mg/L. Case 2 (UM) showed no response despite maximum dosing (400 mg/day) and highest plasma level (28.22 mg/L), suggesting pharmacokinetic failure at biophase level. Case 5 (PM) experienced dose-limiting toxicity at 100 mg/day in complex polypharmacy context with suspected drug-drug-gene interaction. These preliminary observations raise the hypothesis that extreme metabolic phenotypes might be associated with differential risk, and integrated PGx-TDM assessment may provide clinically actionable information beyond plasma monitoring alone.

## Discussion

This exploratory case series, drawn from a source cohort of 91 patients with focal drug-resistant epilepsy (from 125 who initiated CNB at our center), describes preliminary observations on the possible relationship between pharmacogenetic profiles and clinical outcomes during CNB therapy. Our institutional strategies—the 100 mg clinical checkpoint and proactive digital communication protocol—contributed to the safe characterization of CNB’s clinical profile. While these observations do not establish causal relationships, they raise the hypothesis that integrating pharmacogenetics and therapeutic drug monitoring may warrant further investigation as a complementary approach to CNB management. Moreove, our study highlights that the clinical response to CNB is not merely a function of dosage but is significantly influenced by the patient’s genetic architecture. The molecular findings and the functional impact of the detected variants were interpreted in the context of plasma drug concentrations and clinical seizure response, so that genotype-derived phenotype calls were evaluated alongside pharmacokinetic and pharmacodynamic evidence.

### Dose refinement and polypharmacy reduction

Cases 1, 3, and 4 demonstrate substantial clinical response at sub-target doses (100–150 mg/day), below the recommended maintenance dose of 200 mg/day per FDA labeling. This aligns with emerging real-world evidence suggesting that some patients may achieve meaningful efficacy during the titration phase, before reaching the standard target dose (Novitskaya et al., 2024; Villani et al., 2022). The BLESS study similarly reported meaningful efficacy at lower doses (Lattanzi et al., 2025), supporting individualized, conservative dosing strategies. It is important to note that according to the Summary of Product Characteristics (SmPC) and FDA labeling, 200 mg/day represents the recommended maintenance (target) dose, not a ‘low dose.’ The 100 mg/day checkpoint in our protocol corresponds to weeks 7–8 of the standard titration schedule. Patients achieving efficacy at this dose represent early responders during the titration phase, which may have implications for individualized dosing strategies.

Case 1—an ultra-refractory patient with lissencephaly achieving sustained seizure freedom at 100 mg over 32 months—highlights the anticipated titration checkpoint’s success. The Normal Metabolizer phenotype, achieved through compensatory balance between reduced CYP2A6 and preserved UGT2B7/CYP2E1 function, may explain why minimal dose proved sufficient. Case 3 illustrates CNB’s high potency allowing de-escalation of concomitant ASMs, including complete VPA discontinuation and significant LTG reduction. Reducing polypharmacy burden minimizes cumulative adverse events, improves cognitive function, reduces drug-drug interactions, and enhances compliance (Lattanzi et al., 2025).

### Pharmacogenetics and non-response: exploring pharmacokinetic from pharmacodynamic failure

Case 2 presents a unique challenge: a non-responder tolerating maximal dose (400 mg/day) without adverse events, with plasma level 28.22 mg/L—the highest in our series. Traditionally, this would be labeled pharmacodynamic resistance. However, PGx analysis revealed a predicted UM phenotype: UGT2B7 Haplotype 4 + Haplotype II (associated with markedly enhanced glucuronidation), CYP2A6*46/*46 (increased oxidative metabolism), and CYP2E1 gene duplication. This combination may theoretically accelerate CNB clearance and reduce biophase concentrations below the effective threshold, although this remains speculative in the absence of direct biophase measurements. We therefore consider a pharmacokinetic contribution to non-response a plausible, hypothesis-generating explanation rather than an established conclusion.

This observation raises important questions about conventional TDM interpretation. While 28.22 mg/L may appear adequate, validated therapeutic ranges for CNB have not been established; current reference values derive from clinical trial populations and may not account for metabolic extremes (Steinhoff et al., 2024). In patients with predicted UM phenotypes, rapid systemic clearance may result in greater peak-to-trough fluctuations, potentially reducing sustained receptor occupancy despite adequate trough levels. Furthemore, the relationship between plasma and brain CNB concentrations has not been characterized across different metabolic phenotypes; enhanced peripheral clearance might or might not affect CNS penetration depending on transporter activity and blood-brain barrier dynamics*.* Alternatively, if brain penetration is preserved despite enhanced peripheral metabolism, Case 2 may represent true pharmacodynamic resistance—intrinsic refractoriness at drug target level. Future investigation of pharmacodynamic genetic variants, such as GABA_A receptor subunit polymorphisms (GABRA1, GABRB2, GABRG2) or sodium channel variants (SCN1A, SCN2A, SCN8A), would be needed to explore this possibility. This case illustrates the potential value of integrated PGx-TDM assessment beyond plasma monitoring alone, and raises the question of whether patients with predicted UM phenotypes might benefit from doses exceeding 400 mg/day—a hypothesis requiring careful safety evaluation in prospective studies.

### Expanding therapeutic phenotype: earlier intervention

Case 4—a patient with shorter drug resistance history achieving seizure freedom—illustrates our institutional learning curve’s critical outcome. The sustained success of our safety protocols provided clinical confidence to expand CNB use to patients with less extreme drug resistance. While initial trials focused on ultra-refractory cohorts (Peña-Ceballos et al., 2023; Villanueva et al., 2023), recent experiences advocate earlier introduction of highly effective ASMs to maximize sustained seizure freedom (Lattanzi et al., 2025). This evolution reflects growing recognition that prolonged uncontrolled epilepsy carries cumulative risks: cognitive decline, psychosocial impairment, injury, SUDEP, and progressive treatment resistance.

### Pharmacogenetic risk and drug-drug-gene interactions

Case 5, characterized by dose-limiting adverse effects (intense vertigo and somnolence) at the low dose of 100 mg/day, illustrates the critical concept of phenoconversion through a drug-drug-gene interaction. While the patient’s CNB level was low (8.16 mg/L), the dose-limiting vertigo and somnolence at 100 mg were hypothesized to be driven by CNB’s inhibition on *CYP2C19*. While N-desmethylclobazam levels were not measured, thereby making the specific metabolic mechanism speculative, the interaction remains biologically plausible given the patient’s underlying *UGT2B7* and *CYP2A6* (*CYP2A6* *18) variants, which suggest a likely reduced-efficiency metabolic phenotype. This case underscores the importance of considering not only the PGx of the primary drug but also the potential for drug-drug-gene interaction where CNB alters the functional metabolic phenotype of co-administered ASMs like CLB.

### Exploratory institutional approach to CNB management

Based on our institutional experience, we describe an exploratory, non-validated approach to CNB management that emerged from the clinical observations in this case series. This framework is not intended as a practice-ready algorithm, but rather as a preliminary conceptual model that requires prospective validation before any clinical implementation.

Our institutional approach evolved through three empirical steps:

Step 1 — Standardized Titration with Early Clinical Assessment: CNB was initiated per official labeling, with a systematic clinical evaluation at the 100 mg/day milestone to assess early efficacy signals and tolerability before further dose escalation. This step is consistent with emerging real-world evidence suggesting that some patients may respond at sub-target doses (Novitskaya et al., 2024; Villani et al., 2022).
Step 2 — Individualized Dose Optimization: In patients achieving seizure freedom or ≥50% seizure reduction at the 100 mg clinical checkpoint, the sub-target dose was maintained and de-escalation of concomitant ASMs was considered. In patients with inadequate response but good tolerability, dose escalation toward the target (200 mg/day) or high dose (up to 400 mg/day) was pursued with TDM monitoring.
Step 3 — Retrospective PGx Analysis for Hypothesis Generation: In selected cases with unexpected outcomes—non-response despite maximum dosing and apparently adequate plasma levels, or dose-limiting toxicity at sub-target doses—retrospective PGx analysis was performed to explore possible pharmacogenetic contributors. In our limited experience, this analysis generated hypotheses regarding possible associations between UM phenotype and non-response (Case 2) and between PM phenotype and dose-limiting toxicity (Case 5). However, these associations remain unvalidated and should not guide clinical decisions outside of research protocols.

We emphasize that this framework represents an exploratory institutional approach derived from five illustrative cases and should not be interpreted as a practice-ready algorithm. Prospective validation in adequately powered, multi-center cohorts is essential before any clinical implementation can be recommended.

### Comparison with existing literature

To our knowledge, this is the first study systematically integrating multi-gene NGS pharmacogenetic profiling with TDM for CNB. Our findings are consistent with emerging real-world evidence demonstrating CNB efficacy at lower doses than pivotal trials (Novitskaya et al., 2024; Villani et al., 2022; Lattanzi et al., 2025). Our observations are consistent with emerging real-world evidence suggesting CNB efficacy at sub-target doses (Novitskaya et al., 2024; Villani et al., 2022; Lattanzi et al., 2025). The possible association between metabolic phenotype and clinical outcome, if confirmed, would align with the broader pharmacogenomics literature, where UM and PM phenotypes have been associated with therapeutic failure and toxicity across multiple drug classes (Relling and Evans, 2015). However, the specific genetic variants identified in our cases have not been previously studied in the context of CNB, and these associations remain preliminary and hypothesis-generating. However, the specific genetic variants identified—UGT2B7 Haplotype 4, CYP2A6*12, CYP2A6*46—have not been previously studied in CNB context, representing novel hypothesis-generating observations warranting validation in larger cohorts.

As an exploratory case series, these observations are hypothesis-generating and cannot establish clinical utility. Pharmacogenetic implementation in epilepsy requires well-curated data relating individual genetic variants to clinical outcomes, replication in independent cohorts, and demonstration of clinical validity and utility before incorporation into practice. The patterns observed in our five cases—while clinically suggestive—require confirmation in larger studies before any causal inferences can be drawn.

### Limitations and future directions

Several limitations must be acknowledged. As a retrospective case series of five purposively selected vignettes from a larger cohort, these observations are hypothesis-generating and require validation in larger, prospective, multi-center trials. The small sample size precludes statistical analysis and limits generalizability. Selection bias is inherent in purposive sampling. Additional limitations include: lack of validated therapeutic range for CNB; absence of nCLB measurement in Case 5; literature-based phenotype translation for genes without CPIC guidelines; and no pharmacodynamic genetic analysis.

Future research should prioritize: (Nakamura et al., 2019): validating UGT2B7, CYP2E1, and CYP2A6 variants as clinically actionable markers in adequately powered cohorts; (Sharma et al., 2021); expanding genetic scope to include drug transporters (ABCB1, SLC family) and pharmacodynamic targets (GABA_A receptor subunits, sodium channel genes); (Perucca et al., 2023); developing integrated PGx-TDM decision support tools; and (Krauss et al., 2020) conducting cost-effectiveness analyses. Finally, a significant limitation in our interpretation of Case 5 is the absence of N-desmethylclobazam measurements, which makes the proposed drug-drug-gene interaction a hypothesis rather than a confirmed explanation. This underscores the necessity of prospective validation where all relevant metabolites are quantified. Despite these limitations, the preliminary associations observed between extreme metabolic phenotypes and clinical outcomes suggest that further investigation of PGx-guided CNB therapy is warranted. Prospective, multi-center studies with adequate sample sizes are needed to determine whether these associations are reproducible and clinically actionable.

## Data Availability

The datasets presented in this study can be found in online repositories. The names of the repository/repositories and accession number(s) can be found in the article/Supplementary Material.
