# Correction: Precision management of cenobamate in drug-resistant epilepsy: integrating pharmacogenetics, therapeutic drug monitoring, and real-world clinical strategies

**DOI:** 10.3389/fphar.2026.1939779

**Published:** 2026-07-28

**Authors:** Giuseppe d'Orsi, Maria Teresa Di Claudio, Alessia Cafaro, Sebastiano Barco, Marta Robustella, Nicolò Locatelli, Antonella Liantonio, Paola Imbrici, Grazia Ciavarella, Antonio Rinaldi, Giuseppe Fania, Umberto Costantino, Massimo Carella, Giuliana Cangemi, Giuseppe Miscio

**Affiliations:** 1 Neurology Unit - Epilepsy Center, Fondazione IRCCS Casa Sollievo Della Sofferenza, San Giovanni Rotond, Italy; 2 Biochemistry, Pharmacology and Newborn Screening Unit, Central Laboratory of Analyses, IRCCS Istituto Giannina Gaslini, Genova, Italy; 3 Department of Pharmacy - Drug Sciences, University of Bari “Aldo Moro”, Bari, Italy; 4 Medicina Trasfusionale e Laboratorio Analisi, Fondazione IRCCS Casa Sollievo Della Sofferenza, San Giovanni Rotondo, Italy; 5 UOC Genetica Medica, Fondazione IRCCS Casa Sollievo Della Sofferenza, San Giovanni Rotondo, Italy

**Keywords:** cenobamate, pharmacogenetics, drug-resistant epilepsy, real-world evidence, therapeutic drug monitoring, precision medicine

The acknowledgements section was missing in the published article. The correct Acknowledgements section is: CYP2A6 was Sanger sequenced and tested for copy number variation by Andrea Gaedigk, PhD and Erin Boone, PhD, Division of Clinical Pharmacology, Toxicology & Therapeutic Innovation, Children’s Mercy Research Institute, Kansas City, MO, USA.

The original version of this article has been updated.

