# Supplementary material for: Precision management of cenobamate in drug-resistant epilepsy: integrating pharmacogenetics, therapeutic drug monitoring, and real-world clinical strategies
Source: Front Pharmacol. 2026 Jun 4;17:1830217. doi: 10.3389/fphar.2026.1830217 (PMC13275425; doi:10.3389/fphar.2026.1830217)
Supplement: Supplementary file 1 [file Table1.docx]

| **Gene** | **Case** | **Genotype / Variant Note** |
| --- | --- | --- |
| UGT2B7 | Case 1 | Haplotype II (c.-900AA) — mildly enhanced promoter activity |
|  | Case 2 | Haplotype 4 + Haplotype II — markedly enhanced glucuronidation |
|  | Case 3 | Haplotype 4 + Haplotype II — enhanced glucuronidation (as Case 2) |
|  | Case 4 | Haplotype I (c.-900GG) — slightly reduced promoter vs Haplotype II; classified NM/IM |
|  | Case 5 | Haplotype I/II (c.-900AG) — mild reduced promoter activity |
| UGT2B4 | Case 1 | c.1374G>A het (Asp458Glu) — relevant only in homozygosity |
|  | Case 2 | No variants of interest |
|  | Case 3 | No variants of interest |
|  | Case 4 | No variants of interest |
|  | Case 5 | No variants associated with altered function |
| CYP2E1 | Case 1 | c.-71T/T — mildly increased transcriptional activity |
|  | Case 2 | Gene duplication + rs2070673 het — homeostatic compensation; mild increase |
|  | Case 3 | No variant of interest |
|  | Case 4 | No variant of interest |
|  | Case 5 | No variant of interest |
| CYP2B6 | Case 1 | *1/*1 — NM per CPIC |
|  | Case 2 | *1/*5 — NM per CPIC |
|  | Case 3 | *1/*1 — NM |
|  | Case 4 | *1/*1 — NM |
|  | Case 5 | *1/*1 — NM |
| CYP2C19 | Case 1 | *2/*2 — PM; complete loss of function |
|  | Case 2 | No significant variants |
|  | Case 3 | No significant variants |
|  | Case 4 | No significant variants |
|  | Case 5 | No genetic variants BUT CYP2C19 functionally inhibited by CNB (phenoconversion) → hypothesized effective PM |
| CYP2A6 | Case 1 | *2/*9 — reduced activity diplotype |
|  | Case 2 | *46/*46 + additional gain-of-expression variants — increased activity |
|  | Case 3 | *12/*12 — near-complete loss of function (hybrid CYP2A6-CYP2A7); <5% residual activity |
|  | Case 4 | *1/*1 — NM |
|  | Case 5 | *18 het (c.1174T>C; p.Tyr392Phe) — reduced enzymatic efficiency |

**Supplementary Table 1: Patient Genotype profiles**. This document provides a detailed summary of the specific genetic variants and genotypes identified for each of the six enzymes analyzed across the clinical cases
