# Supplementary material for: Precision management of cenobamate in drug-resistant epilepsy: integrating pharmacogenetics, therapeutic drug monitoring, and real-world clinical strategies
Source: Front Pharmacol. 2026 Jun 4;17:1830217. doi: 10.3389/fphar.2026.1830217 (PMC13275425; doi:10.3389/fphar.2026.1830217)
Supplement: Supplementary file 2 [file Table2.docx]

**Supplementary Table 2: Composite Phenotype derivation.** This table integrates allele functional assignments, pathway weights, and hypothesized enzymatic activity to derive the overall composite metabolizer phenotype for the patients.

| Patient (Composite  Phenotype) | Gene | Allele / Haplotype  (HGVS / rsID / Star Allele) | Allele Function Assigned | Exploratory and hypothesized Activity | Pathway  Weight  (CNB) | Evidence Framework  (Guideline / Literature) | Composite Phenotype Derivation  (first row per patient; see footnotes) |
| --- | --- | --- | --- | --- | --- | --- | --- |
| Case 1 (NM) | UGT2B7 | Haplotype II – homozygous c.-900A/A (rs7438135); coding variants without functional effect | Normal (Hap II: full promoter activity; reference for population) | Normal promoter activity | Primary | Hu et al. 2014 | NM: Primary pathway (UGT2B7 Hap II) fully functional → weighted net activity balanced. CYP2A6 reduced (minor pathway) offset by normal UGT2B7/UGT2B4/CYP2E1. CYP2C19 PM (no function): negligible CNB impact as confirmed by TDM (12.28 mg/L) and SSF at 100 mg — consistent with minor pathway as per drug label (Refs 1,11–12). Genotype-to-phenotype call follows CPIC framework (Tibben et al.) and CAP/AMP variant annotation principles (Ji et al.) |
|  | UGT2B4 | c.1374G>A (p.Asp458Glu, rs13119049) –heterozygous | Normal (heterozygous effect only in homozygosity) | Basal | Secondary | Yong et al. 2011 |  |
|  | CYP2E1 | c.-71G>T (rs6413420) – homozygous (5′-UTR) | Mildly increased transcription | Mildly increased transcriptional activity | Secondary | Fairbrother et al. 1998 |  |
|  | CYP2A6 | *2/*9: c.-48T>G (rs28399433) het + c.479T>A p.Leu160His (rs1801272) het | Decreased (reduced-activity diplotype, both alleles impaired) | Reduced activity diplotype | Minor | Tanner & Tyndale 2017; Oscarson 2002 |  |
|  | CYP2B6 | *1/*1 – NM per CPIC | Normal | NM per CPIC | Secondary | https://www.clinpgx.org/gene/PA123/haplotype; Desta et al. 2021; Ji et al. 2025 |  |
|  | CYP2C19 | *2/*2 (homozygous loss-of-function; PM) | No function (complete CYP2C19 loss) | Complete loss of function | Minor | https://www.clinpgx.org/gene/PA124/haplotype |  |

Gene-by-gene pharmacogenetic variant calls, allele function assignments, normalized activity values, pathway weights, and composite metabolizer phenotype derivation for five cenobamate-treated patients. Nomenclature follows CPIC/PharmVar star allele definitions and HGVS standards. Phenotype predictions for CPIC-curated genes follow validated CPIC/CAP MOL.36155 standards. Assessments for UGT2B7, UGT2B4, and CYP2E1 are exploratory and based on published functional literature. Composite phenotype derivation follows the CNB framework (Tibben et al., Am J Hum Genet, 2025). Reporting standards per Ji et al. (Arch Pathol Lab Med, 2025; doi: 10.5858/arpa.2025-0179-CP) and CAP MOL.49570.

| Patient (Composite  Phenotype) | Gene | Allele / Haplotype  (HGVS / rsID / Star Allele) | Allele Function Assigned | Exploratory and hypothesized Activity | Pathway  Weight  (CNB) | Evidence Framework  (Guideline / Literature) | Composite Phenotype Derivation  (first row per patient; see footnotes) |
| --- | --- | --- | --- | --- | --- | --- | --- |
| Case 2 (UM) | UGT2B7 | Haplotype 4 [c.722-314A>G (rs62298861); c.735A>G p.Tyr245 (rs28365062); c.1062C>T p.Tyr354 (rs4348159)] het + Haplotype II c.-900A/A | Increased (Hap 4 ↑ UGT2B7 expression and activity in vivo/in vitro) | Markedly enhanced glucuronidation | Primary | Innocenti 2008; Kwara 2009; Hu 2014 | UM: Both primary (UGT2B7 Hap 4, markedly increased activity ) and minor (CYP2A6 *46/*46, activity) show increased function. Hypothesized weighted composite = UM. Clinical correlate: non-responder at 400 mg/day, TDM CNB 28.22 mg/L. Phenotype call supported by CPIC activity score binning logic (Tibben 2025) and HGVS/star allele reporting standards (Ji et al.; CAP MOL.36155). |
|  | UGT2B4 | No functionally relevant variants detected | Normal | Basal | Secondary | Yong 2011 |  |
|  | CYP2E1 | Whole-gene CNV duplication (×3 copies); c.-333T>A (rs2070673) het in promoter | Mild increased (gene CNV with homeostatic compensation – no net overexpression; promoter variant) | Mild increase activity | Secondary | Tremmel 2016; Huang 2012 |  |
|  | CYP2A6 | *46/*46 – homozygous 3′ gene conversion (CYP2A6→CYP2A7) | Increased (homozygous *46 + additional expression-increasing variants) | Increased activity | Minor | Langlois 2024; Han 2012 |  |
|  | CYP2B6 | *1/*5 – NM per CPIC | Normal | NM per CPIC | Secondary | https://www.clinpgx.org/gene/PA123/haplotype; Desta et al. 2021; Ji et al. 2025 |  |
|  | CYP2C19 | *1/*1 – NM per CPIC | Normal | NM per CPIC | Minor | https://www.clinpgx.org/gene/PA124/haplotype |  |

| Patient (Composite  Phenotype) | Gene | Allele / Haplotype  (HGVS / rsID / Star Allele) | Allele Function Assigned | Exploratory and hypothesized Activity | Pathway  Weight  (CNB) | Evidence Framework  (Guideline / Literature) | Composite Phenotype Derivation  (first row per patient; see footnotes) |
| --- | --- | --- | --- | --- | --- | --- | --- |
| Case 3 (NM) | UGT2B7 | Haplotype 4 (same variants as Case 2, heterozygous) + Haplotype II c.-900A/A | Increased (Hap 4 ↑ UGT2B7 expression and activity in vivo/in vitro) | Markedly enhanced glucuronidation | Primary | Innocenti 2008; Kwara 2009; Hu 2014 | UGT2B7 Hap4 (increased activity) and CYP2A6 12/12 (near-zero loss of function): divergent gene-level functional effects. The integrated pathway-weighted exploratory interpretation is consistent with NM phenotype, corroborated by an adequate therapeutic response in the absence of treatment-related toxicity at 150 mg/day (TDM: CNB 14.84 mg/L). |
|  | UGT2B4 | No functionally relevant variants detected | Normal | Basal | Secondary | Yong 2011 |  |
|  | CYP2E1 | No variant of interest | Normal | Basal | Secondary | — |  |
|  | CYP2A6 | *12/*12 – homozygous CYP2A6-CYP2A7 hybrid gene (3′ exon conversion exons 3–9) | No function / severely decreased (*12/*12 hybrid – near-complete loss; residual activity retained) | Near-complete loss of function very low residual activity | Minor | Oscarson 2002; Fukami 2005 |  |
|  | CYP2B6 | *1/*1 – NM per CPIC | Normal | NM per CPIC | Secondary | https://www.clinpgx.org/gene/PA123/haplotype; Desta et al. 2021; Ji et al. 2025 |  |
|  | CYP2C19 | *1/*1 – NM per CPIC | Normal | NM per CPIC | Minor | https://www.clinpgx.org/gene/PA124/haplotype |  |

| Patient (Composite  Phenotype) | Gene | Allele / Haplotype  (HGVS / rsID / Star Allele) | Allele Function Assigned | Exploratory and hypothesized Activity | Pathway  Weight  (CNB) | Evidence Framework  (Guideline / Literature) | Composite Phenotype Derivation  (first row per patient; see footnotes) |
| --- | --- | --- | --- | --- | --- | --- | --- |
| Case 4 (NM) | UGT2B7 | Haplotype I – homozygous c.-900G/G (rs7438135); reduced promoter activity Vs Haplotype II | Decreased (Hap I: reduced promoter activity vs Hap II; lowest among haplotypes) | Reduced promoter activity | Primary | Hu 2014 | Reduced activity variant. UGT2B7 Hap I below reference. Least polymorphic patient overall. Consistent with SSF at 100 mg/day. |
|  | UGT2B4 | No functionally relevant variants detected | Normal | Basal | Secondary | Yong 2011 |  |
|  | CYP2E1 | No variant of interest | Normal | Basal | Secondary | — |  |
|  | CYP2A6 | *1/*1 – NM | Normal | NM per CPIC | Minor | Tanner & Tyndale 2017 |  |
|  | CYP2B6 | *1/*1 – NM | Normal | NM per CPIC | Secondary | https://www.clinpgx.org/gene/PA123/haplotype; Desta et al. 2021; Ji et al. 2025 |  |
|  | CYP2C19 | *1/*1 – NM | Normal | NM per CPIC | Minor | https://www.clinpgx.org/gene/PA124/haplotype |  |

| Patient (Composite  Phenotype) | Gene | Allele / Haplotype  (HGVS / rsID / Star Allele) | Allele Function Assigned | Exploratory and hypothesized Activity | Pathway  Weight  (CNB) | Evidence Framework  (Guideline / Literature) | Composite Phenotype Derivation  (first row per patient; see footnotes) |
| --- | --- | --- | --- | --- | --- | --- | --- |
| Case 5 (Exploratory IM / Reduced  Efficiency) | UGT2B7 | Haplotype I/II – heterozygous c.-900A/G; mildly reduced promoter activity vs Haplotype II | Normal–Decreased Vs Hap II | Mild reduction | Primary | Hu 2014 | Hypothesized Reduced-efficiency: UGT2B7 Hap I/II + CYP2A6 *1/*18 → combined reduced CNB clearance. Drug-drug-gene interaction: hypothesized CNB-mediated CYP2C19 inhibition → N-desmethylclobazam accumulation (CLB active metabolite) → toxicity at 100 mg/day. Composite phenotype reflects both primary pathway reduction and clinically significant DDGi per CPIC phenoconversion principles. |
|  | UGT2B4 | No functionally relevant variants detected | Normal | Basal | Secondary | Yong 2011 |  |
|  | CYP2E1 | No functionally relevant variants detected | Normal | Basal | Secondary | — |  |
|  | CYP2A6 | *1/*18 heterozygous: c.1174T>C p.Tyr392Phe (rs1809810) ; IM | *1/*18: substrate recognition impairment, reduced enzymatic efficiency | IM/Reduced enzymatic efficiency | Minor | Tanner & Tyndale 2017; Fukami 2005; Han 2012 |  |
|  | CYP2B6 | *1/*1 – NM | Normal | NM per CPIC | Secondary | https://www.clinpgx.org/gene/PA123/haplotype; Desta et al. 2021; Ji et al. 2025 |  |
|  | CYP2C19 | *1/*1 – NM | Normal | NM per CPIC | Minor | https://www.clinpgx.org/gene/PA124/haplotype |  |

Methodological Notes

1 Diplotype assignment and phasing. Diplotype assignments for all CPIC/PharmVar star allele–curated genes (CYP2A6, CYP2B6, CYP2C19) were assigned putatively/empirically based on the most likely allele combinations derived from population frequency databases (PharmVar; pharmvar.org), in the absence of long-read sequencing phasing data. This approach is consistent with current standard practice as described in Ji et al. and the CPIC/PharmVar framework.

2 Genes without CPIC/PharmVar star allele systems (UGT2B7, UGT2B4, CYP2E1). No assigned star allele nomenclature is currently available for UGT2B7, UGT2B4, or CYP2E1. Variants are reported in HGVS format based on the following reference transcripts: UGT2B7 — NM_001074.3; UGT2B4 — NM_001080.3; CYP2E1 — NM_000773.4. rsID cross-references are provided per entry. Allele function assignments represent investigational/exploratory classifications based on published functional literature (primary references cited per entry) and are NOT currently endorsed by CPIC or PharmVar as standardized phenotype categories. In addition, according to the CPIC framework described (Ref 35), the general rule is that clinical allele function assignments are "drug-agnostic" (independent of the drug). Once a clinical function is established for an allele—even if the supporting data comes from a specific drug—that assigned function is used to map phenotypes for all drugs metabolized by that enzyme. CPIC prefers “drug-agnostic” assignments to promote standardization and interoperability of pharmacogenetic results across different clinical settings.

3 Absence of reportable variants. "No functionally relevant variants detected" or "No variant of interest" indicate that no variants within the pre-specified target regions reached the analytical call threshold of this assay. This does not exclude the presence of variants outside the interrogated genomic regions, rare structural variants, or alleles absent from current PharmVar/CPIC databases.

4 Empirical diplotype assignment for star allele genes. For CYP2A6, CYP2B6, and CYP2C19, haplotype calling and diplotype assignment were performed empirically using PharmVar/CPIC databases. Standard short-read NGS platforms cannot phase variants across alleles without additional long-range PCR or long-read sequencing. Diplotypes represent the most probable assignment; alternative rare combinations cannot be formally excluded. Phenotype classification is reported for the assigned diplotype only. In accordance with Ji et al. (Ref 34), the most common diplotype is assigned by default when multiple configurations are possible. For non star alleles assigned genes (UGTs) ULaval Official UGTs Database was consulted (https://www.pharmacogenomics.pha.ulaval.ca/ugt-alleles-nomenclature/).

5 Terminology: The term "wild-type" has been replaced by the PharmVar-designated reference allele (*1) in all entries, per current pharmacogenomic nomenclature recommendations (PharmVar; CPIC). The *1/*1 diplotype corresponds to Normal Metabolizer (NM) status for CYP2A6, CYP2B6, and CYP2C19 per validated CPIC phenotype translation tables.

6 Complex structural variants — CYP2A6 gene conversions (Cases 2 and 3). The CYP2A6→CYP2A7 gene conversion alleles (*46/*46, Case 2; *12/*12, Case 3) were identified by NGS-based structural variant analysis. These represent complex genomic rearrangements with partial homology to the CYP2A7 pseudogene. Orthogonal confirmation by Sanger Sequencing and long-read sequencing. Current assignments are consistent with published allele definitions (PharmVar; Oscarson 2002; Fukami 2005; Langlois 2024).

7 UGT2B7 Haplotype I activity classification (Cases 4 and 5). UGT2B7 Haplotype I (c.-900G/G, rs7438135) shows modestly reduced promoter activity relative to Haplotype II (c.-900A/A) in published in vitro studies (Hu et al., 2014). However, prospective pharmacokinetic outcome data validating clinical significance are lacking, and no CPIC/PharmVar consensus phenotype classification exists. "Decreased/Normal" reflects this functional uncertainty and should not be equated with a validated CPIC Intermediate or Poor Metabolizer classification. This assignment is exploratory and subject to revision.

CAP MOL.49570 — Mandatory Disclaimer (applicable to all cases). In accordance with CAP MOL.49570, this report includes a summary of the analytical methods, the gene regions and variants interrogated, and the analytic interpretation for each case. The predicted composite metabolizer phenotype is derived solely from the genetic variants detected by the assay. Undetected genetic variants (outside interrogated regions or below the analytical call threshold) and non-genetic factors — including drug-drug interactions, hepatic/renal function, co-medications (especially UGT2B7 inhibitors and CYP2A6 inhibitors), and comorbidities — may independently or synergistically alter the actual drug response phenotype and are not captured by this analysis. This caveat is of particular clinical relevance for Case 3 (opposing pathway effects) and Case 5 hypothesized PM-Phenoconversion.
